# On the challenge of assessing dynamic cerebral autoregulation

**DOI:** 10.1113/EP091703

**Published:** 2024-05-07

**Authors:** Patrice Brassard, Marc‐Antoine Roy, Lawrence Labrecque, Jonathan D. Smirl

**Affiliations:** ^1^ Department of Kinesiology, Faculty of Medicine Université Laval Québec Canada; ^2^ Research center of the Institut universitaire de cardiologie et de pneumologie de Québec Québec Canada; ^3^ Sport Injury Prevention Research Centre, Faculty of Kinesiology University of Calgary Calgary Alberta Canada; ^4^ Cerebrovascular Concussion Laboratory, Faculty of Kinesiology University of Calgary Calgary Alberta Canada; ^5^ Hotchkiss Brain Institute University of Calgary Calgary Alberta Canada

**Keywords:** cerebral pressure‐flow relationship, directional sensitivity, dynamic cerebral autoregulation, transfer function analysis

## INTRODUCTION

Connections link a sequence of three related research papers. The central article which links the other two papers has been published in *Experimental Physiology*. In a Connections article, an author (or authors) of the central article outlines its principal novel findings, tracing how they were influenced by the first article and how the central article has contributed to the developments made in the third article. The author(s) may also speculate on the direction of future research in the field. Connections articles aim to set the research in a wide context.

Dynamic cerebral autoregulation (dCA) represents the ability of the cerebrovasculature to respond to transient changes in arterial blood pressure (ABP). The quantification of dCA remains a complex endeavour considering that a gold‐standard to examine the black‐box nature of this entity does not exist. Over the years, investigators have applied numerous methods and approaches and used diverse metrics to quantify dCA based on a single change, or oscillations (spontaneous/forced), in ABP (reviewed in Brassard et al., 2023). Unfortunately, limited research has included multi‐method strategies or completed comparisons between analytical approaches when assessing dCA. Distinctive stressors (e.g., spontaneous vs. forced ABP oscillation; increase vs. decrease in ABP) can differently engage and challenge the regulatory mechanisms associated with dCA, thus providing different physiological information. As most dCA metrics appear unrelated to each other, comparing findings across different methods and analytical strategies is challenging.

To help illustrate the challenge of comparing and interpreting dCA findings using different stresses and analytical methods, we will use results from three dCA reports focusing on the influence of different exercise training modalities (i.e., endurance and resistance exercise training) in young healthy individuals. Endurance and resistance exercise induce distinct physiological adaptations, likely because of different haemodynamic responses induced by these exercise training types. Generally, habitual exercise training leads to beneficial cerebrovascular function adaptations, such as resting cerebral perfusion and cerebrovascular carbon dioxide reactivity. However, existing evidence also shows an absence of impact, or even detrimental effects, of habitual exercise training on some aspects of cerebrovascular function, such as dCA. These equivocal findings may partly be the consequence of differences in exercise training types completed by participants.

Despite the existing modality‐dependent cerebrovascular responses, findings from a recent cross‐sectional study suggest the absence of an endurance or resistance habitual exercise effect on dCA metrics derived from transfer function analysis (TFA) on forced ABP and cerebral blood velocity (CBv: a surrogate of cerebral blood flow) induced by repeated squat–stands performed at 0.05 and 0.10 Hz (Perry et al., 2019). The objective of TFA, a popular method to quantify dCA, is to estimate variables reflecting the dynamic behaviour of dCA, supposing the latter represents a linear control system between the input (ABP) and the output (CBv). TFA metrics are: coherence (i.e., fraction of the ABP linearly related to CBv), gain (i.e., CBv amplitude change for a given ABP change), and phase (i.e., timing difference of ABP and CBv waveforms). Spontaneous (e.g., at rest) and driven ABP oscillations are utilized for dCA quantification using TFA. Spontaneous ABP fluctuations are attractive for those who want to quantify dCA in populations in whom it is not possible, or safe, to force ABP oscillations of larger amplitude (e.g. patients with cerebrovascular diseases). However, the limited amplitude of spontaneous oscillations (i.e., low signal‐to‐noise ratio) will usually lead to less reliable and reproducible estimations of dCA metrics when using TFA. Techniques, such as repeated squat–stands, are thus utilized to augment the input power (i.e., ABP) and enhance the linear interpretability and reproducibility of TFA metrics. TFA has shown the cerebral vessels act as a high‐pass filter, which means oscillations slower than 0.20 Hz are dampened and oscillations above 0.20 Hz pass through unimpeded. The two frequencies often used in the dCA literature when forcing ABP to large amplitude (0.05 and 0.10 Hz) are included in the frequency bands where dCA is thought to have the most important influence on the cerebral pressure–flow dynamics (historically, these frequency bands being 0.02–0.07 Hz for the very low frequency and 0.07−0.20 Hz for the low frequency). Also, these two frequencies are most prevalent in the literature and could reveal information about potential mechanisms. In their study, Perry et al. (2019) reported a non‐significant trend for lowered TFA phase with resistance‐trained individuals, compared to endurance‐trained and sedentary individuals, suggesting no clear impact of habitual exercise modality on dCA. Do these findings necessarily mean habitual endurance and resistance exercise cannot influence dCA?

One must consider TFA assumes dCA responses to be linear and symmetric, which is not necessarily the case. For instance, accumulating evidence clearly suggests cerebral vessels react differently to increases, in comparison with decreases, in ABP. Specifically, elevations in CBv are attenuated when ABP increases. This phenomenon has been reported using steady‐state changes, as well as spontaneous and forced ABP oscillations. However, not all dCA analytical approaches take the ABP direction change into consideration (TFA for example). Our group has recently suggested the utilization of a directional sensitivity metric on forced ABP and CBv oscillations induced through a non‐pharmacological approach (i.e., repeated squat–stands at 0.05 and 0.10 Hz) to examine the cerebral pressure–flow relationship when ABP increases and decreases. In a series of studies using this directional sensitivity metric (detailed below), we have reported attenuated increases in middle cerebral artery mean blood velocity (MCAv) when mean arterial pressure (MAP) is forced at the higher repeated squat–stands frequency (i.e., 0.10 Hz: indicative of sympathetic tone associated with Mayer waves) only.

In the same cohort of endurance‐ and resistance‐trained individuals used by Perry et al. (2019), we subsequently quantified dCA using our proposed directional sensitivity metric (Roy et al., 2022). Specifically, we calculated absolute (ΔMCAv_T_/ΔMAP_T_) and relative (%MCAv_T_/%MAP_T_) changes with respect to transition time intervals of both variables to calculate a time‐adjusted ratio in each MAP direction, averaged over a 5‐min series of repeated squat–stands. Using this analysis, ΔMCAv_T_/ΔMAP_T_ and %MCAv_T_/%MAP_T_ were lower during ABP increases in comparison to ABP decreases for sedentary and endurance‐trained individuals, but not for resistance‐trained participants at 0.10 Hz (Roy et al., 2022). These ratios were not different for sedentary, endurance‐trained and resistance‐trained individuals at 0.05 Hz, as previously reported by our group in healthy individuals. These results suggest exercise training modality influences dCA directionality specifically during 0.10 Hz repeated squat–stands in sedentary and endurance‐trained participants, but not in resistance‐trained individuals. The presence of dCA directional sensitivity suggests the cerebrovasculature selectively defends the microcirculation from overperfusion during transient ABP surges. The absence of such a hysteresis‐like pattern in resistance‐trained individuals could be interpreted as an attenuated ability of the cerebrovasculature to react to ABP increases, or alternatively, an improved ability of the cerebrovasculature to react to ABP decreases, compared to sedentary and endurance‐trained individuals. Considering TFA did not provide clear differences in dCA between the same three groups (Perry et al., 2019), this directional sensitivity analysis may represent a more sensitive method than TFA to detect cerebral haemodynamic changes.

**Connected Articles**

- Thomas, H. J., Marsh, C. E., Naylor, L. H., Ainslie, P. N., Smith, K. J., Carter, H. H. & Green, D. J. (2021). Resistance, but not endurance exercise training, induces changes in cerebrovascular function in healthy young subjects. *American Journal of Physiology. Heart and Circulatory Physiology* **321,** H881–H892.
- Roy, M. A., Labrecque, L., Perry, B. G., Korad, S., Smirl, J. D. & Brassard, P. (2022). Directional sensitivity of the cerebral pressure–flow relationship in young healthy individuals trained in endurance and resistance exercise. *Experimental Physiology* **107,** 299–311.
- Panerai, R. B., Barnes, S. C., Batterham, A. P., Robinson, T. G. & Haunton, V. J. (2023). Directional sensitivity of dynamic cerebral autoregulation during spontaneous fluctuations in arterial blood pressure at rest. *Journal of Cerebral Blood Flow & Metabolism* **43,** 552–564.

These directional sensitivity findings are in sharp contrast with results from a recent longitudinal study, which examined the impact of endurance or resistance training on dCA quantified using TFA on spontaneous oscillations (Thomas et al., 2021). In this cross‐over design where 68 young, healthy participants were randomized to complete 3 months of each exercise modality, TFA metrics were not different following endurance and resistance exercise (Thomas et al., 2021). These findings are comparable to those of Perry et al. (2019), who used forced ABP oscillations. As previously mentioned, driven oscillations offer a greater signal‐to‐noise ratio and coherence, in addition to improved reproducibility with repeated squat–stands. Accordingly, forced ABP oscillations may represent a more robust stressor for quantifying the linear aspect of dCA via TFA.

Although our directional sensitivity metric seems promising to quantify the dynamic cerebral pressure–flow relationship, further research is warranted in different experimental conditions and clinical/pathological populations to flesh out the ability to use this approach and the key logistical aspects. Considering our double‐ratio calculation has not been validated for its ability to truly reflect cerebral blood flow regulation, additional work will be necessary to examine whether, for instance, this directional sensitivity metric is influenced by hypercapnia, hypoxia and hyperthermia, or other conditions, such as healthy ageing, cardiovascular and cerebrovascular diseases. Interestingly, other analytical methods exist to characterize the directional sensitivity of the cerebral pressure–flow relationship. For example, previous work, using the repeated squat–stand model performed only at 0.05 Hz described a better dCA response during transient ABP increases, using the autoregulatory index. Panerai et al. (2023) also proposed a new method based on autoregressive‐moving average models dividing the ABP signal into two components—first including the beat‐to‐beat positive derivative information, then the corresponding negative derivative time‐series. Using this approach and contrary to our findings, Panerai et al. (2023) reported the presence of dCA directional sensitivity during ABP oscillations induced at 0.05 Hz using repeated squat–stands. One advantage of this analytical method is it can be applied not only to large transient ABP changes, as with our directional sensitivity method, but also to the relatively small spontaneous ABP oscillations (Panerai et al., 2023). Discrepancies between our findings (Roy et al., 2022) and those from Panerai et al. (2023) at 0.05 Hz repeated squat–stands could be related to methodological differences used to drive ABP oscillations. For instance, the depth of squat was different between our study (participants went to a 45‐degree knee flexion angle) (Roy et al., 2022) and Panerai et al.’s study (participants squatted down as low as they felt able) (Panerai et al., 2023). Alternatively, Panerai et al. pointed out our metric does not take into consideration the phase difference between CBv and ABP (which tends to be higher with 0.05 Hz, in comparison with 0.10 Hz oscillations) (Panerai et al., 2023). They also argue how our sample size (*n* = 12/group) could have been too low, by providing a bootstrap procedure to evaluate the number of participants needed to identify a significant difference in directional sensitivity using their model (critical number: *n* = 24 for repeated squat–stands) (Panerai et al., 2023). Both of these could be reasons why we have not detected directional sensitivity with our analytical method at 0.05 Hz. A next logical step would be to compare these two different dCA directional sensitivity analyses within the same participants performing 0.05 and 0.10 Hz repeated squat–stands.

As one can appreciate, quantifying dCA in the same population (in that case, endurance‐trained, resistance‐trained and sedentary individuals) using different stresses (spontaneous and forced ABP oscillations) and different analytical approaches (TFA and directional sensitivity analysis) can lead to different interpretations. Also, while findings from Thomas et al. (2021) suggest endurance and resistance training do not influence dCA, the utilization of TFA using spontaneous oscillations may not represent the optimal strategy knowing the high variability and poor reproducibility of spontaneous TFA metrics. Finally, although utilization of forced ABP oscillations improves the linear interpretability of TFA, searching for alternatives to TFA is crucial considering the latter does not take the ABP direction change into consideration, while clear evidence exists to support dCA directional sensitivity. We are still in the early stages of directional sensitivity assessment, and here again, different analytical approaches using the same method to force ABP oscillations can lead to different findings (i.e., 0.05 Hz repeated squat–stands). Continued efforts are thus needed to find the best metric, or more likely a collection across several metrics using different physiological stresses, to adequately assess dCA and improve the physiological interpretation of the cerebral pressure–flow relationship.

## AUTHOR CONTRIBUTIONS

All authors have read and approved the final version of this manuscript and agree to be accountable for all aspects of the work in ensuring that questions related to the accuracy or integrity of any part of the work are appropriately investigated and resolved. All persons designated as authors qualify for authorship, and all those who qualify for authorship are listed.

## CONFLICT OF INTEREST

The authors declare no conflicts of interest.

## FUNDING INFORMATION

No funding was received for this work.
